# Immunogenic cancer cell death selectively induced by near infrared photoimmunotherapy initiates host tumor immunity

**DOI:** 10.18632/oncotarget.14425

**Published:** 2017-01-02

**Authors:** Mikako Ogawa, Yusuke Tomita, Yuko Nakamura, Min-Jung Lee, Sunmin Lee, Saori Tomita, Tadanobu Nagaya, Kazuhide Sato, Toyohiko Yamauchi, Hidenao Iwai, Abhishek Kumar, Timothy Haystead, Hari Shroff, Peter L. Choyke, Jane B. Trepel, Hisataka Kobayashi

**Affiliations:** ^1^ Medical Photonics Research Center, Hamamatsu University School of Medicine, Hamamatsu 431-3192, Japan; ^2^ Developmental Therapeutics Branch, Center for Cancer Research, National Cancer Institute, NIH, Bethesda, MD 20892, USA; ^3^ Molecular Imaging Program, Center for Cancer Research, National Cancer Institute, NIH, Bethesda, MD 20892, USA; ^4^ Central Research Laboratory, Hamamatsu Photonics K. K., Hamamatsu 434-8601, Japan; ^5^ Section on High Resolution Optical Imaging, NIBIB/NIH, Bethesda, MD 20892, USA; ^6^ Department of Pharmacology and Cancer Biology, Duke University, Durham, NC 27710, USA; ^7^ Laboratory for Bioanalysis and Molecular Imaging, Graduate School of Pharmaceutical Sciences, Hokkaido University, Sapporo 060-0812, Japan

**Keywords:** near infrared photoimmunotherapy, immunogenic cell death

## Abstract

Immunogenic cell death (ICD) is a form of cell death that activates an adaptive immune response against dead-cell-associated antigens. Cancer cells killed via ICD can elicit antitumor immunity. ICD is efficiently induced by near-infrared photo-immunotherapy (NIR-PIT) that selectively kills target-cells on which antibody-photoabsorber conjugates bind and are activated by NIR light exposure. Advanced live cell microscopies showed that NIR-PIT caused rapid and irreversible damage to the cell membrane function leading to swelling and bursting, releasing intracellular components due to the influx of water into the cell. The process also induces relocation of ICD bio markers including calreticulin, Hsp70 and Hsp90 to the cell surface and the rapid release of immunogenic signals including ATP and HMGB1 followed by maturation of immature dendritic cells. Thus, NIR-PIT is a therapy that kills tumor cells by ICD, eliciting a host immune response against tumor.

## INTRODUCTION

Immunogenic cell death (ICD), which involves changes in the composition of the cell surface and release of soluble mediators, was initially described by Zitvogel and Kroemer *et al* [1–3]. ICD relies on the generation of immunogenic signals induced by a variety of stimuli, including damage-associated molecular patterns (DAMPs) such as the endoplasmic reticulum (ER) chaperone calreticulin, ATP, high mobility group box 1 (HMGB1), heat shock protein (Hsp)70, and Hsp90 [4, 5]. These signals activate dendritic cells (DCs) to stimulate the presentation of tumor-antigens to T-cells. However, most anticancer drugs cause an apoptotic cell death which is tolerogenic and does not elicit immune responses specific for dead cell-associated antigens and therefore, ICD, a potentially useful ally, plays little role in most cancer treatments [4, 6].

Near infrared photoimmunotherapy (NIR-PIT) is a new method of treating cancers by first exposing them to an antibody-photosensitizer conjugate (APC) consisting of an antibody directed at a cell surface antigen overexpressed on the plasma membrane and a photo-activated silica-phthalocyanine (IRDye700DX: IR700) dye [7]. A phase I study of an antibody conjugate consisting of cetuximab (anti-HER1 antibody) linked to IR700, for the treatment of inoperable head and neck cancers is ongoing (NCT02422979). NIR-PIT is unique in that it appears to specifically kill target cells while leaving intact adjacent cells not expressing the antigen [8–11]. The APC binds to cells expressing antigen and after NIR light exposure (690 nm), induces highly selective necrotic cancer cell death with immediately adjacent non-target expressing cells suffering no toxic effects [12] [7]. Microscopy during NIR-PIT reveals rapid bleb formation on the cell membrane within minutes of exposure to light [8].

In this study, we have performed biophysical and immunologic analyses of the events associated with necrotic cell death induced by NIR-PIT. Dynamic morphological changes after NIR-PIT were investigated using three dimensional dynamic low coherence quantitative phase microscopy (3D LC-QPM) [13, 14], which is based on light scattering at the lipid bilayer, and dual-view inverted selective plane illumination microscopy (diSPIM) [15, 16], which uses light-sheet microscopy to follow dynamic changes in fluorescently labeled targets. Additionally, cell membrane permeability was studied on 3D LC-QPM. Finally, we show that NIR-PIT rapidly induces the cardinal signs of ICD, and that NIR-PIT-killed tumor cells induce the maturation of dendritic cells (DCs) suggesting NIR-PIT inducts host antitumor immunity against NIR-PIT-killed tumor cells. These findings can explain superior therapeutic effects of NIR-PIT to cancer in immunocompetent mice or in patients enrolled in an ongoing first-in-human clinical trial compared with that in athymic mice.

## RESULTS

### Rapid increases in cell volume and cell bursting are induced by NIR-PIT

**Figure 1:**
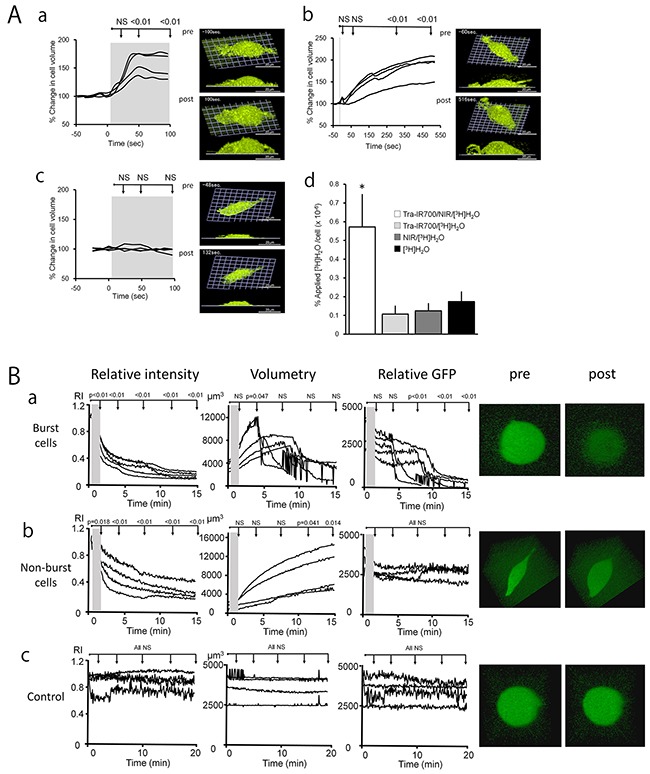
The cells swelled during NIR-PIT **A. a-c**, The time course of % change in cell volume during NIR-PIT calculated by 3D LC-QPM and representative images pre- and post- treatment. 3T3-HER2 cells were treated with Tra-IR700 for 24 hr before 3D LC-QPM observation. The gray area in each graph represents the NIR light exposure duration (**a:** continuous exposure, b: 5-sec exposure, c: continuous exposure in 50 mM dextran). Cells began to swell shortly after exposure to NIR light excitation and reached a maximum within 1 min (**a**). Cell swelling was observed even after only 5-sec exposure of NIR light (b). Cell swelling was not observed after NIR light exposure in 50 mM dextran (**c**). **d**, 3T3-HER2 cells were incubated in culture medium with [^3^H]H_2_O to investigate the water flow-in during NIR-PIT. Uptake of [^3^H]H_2_O was significantly higher in NIR-PIT treated cells compared to non-treated cells (*P* < 0.05). **B**. The time course change in GFP concentration, cell volume, and total GFP amount in cells during NIR-PIT calculated from diSPIM imaging, and representative images pre- and post- treatment. 3T3-HER2 cells were treated with Tra-IR700 for 6 hr before diSPIM observation. The gray area in each graph represents the NIR light exposure duration (**a**, **b:** 1-min exposure, **c:** no exposure). The cells began to swell shortly after exposure to NIR light excitation and half of them burst during observation (**a**) and half of them continued swelling even 15-min after exposure of NIR light (**b**). Control cells with no NIR exposure did not show swelling or decreasing concentration of GFP (**c**). The difference of each parameter at immediately after NIR light exposure, 1, 5, 10, 15, 20, 25 and 30 min after NIR-PIT compared to the starting value, respectively. Differences of *P* < 0.05 were considered significant.

The dynamic 3D LC-QPM imaging showed that Tra-IR700 treated 3T3-HER2 cells began to swell shortly after exposure to NIR, and reached a maximum volume within 1 min after continuous light exposure (Figure 1Aa, Supplementary Video 1). In order to visualize the rapid cell swelling during the continuous light exposure, we also imaged 3D cell morphology at shorter temporal intervals of 3.6 sec (17 slices, scanning depth = 5.6 μm), besides the 3D imaging for volumetry. The cell swelling was observed even after only 5-sec exposure of NIR light (Figure 1Ab, Supplementary Video 2). That is, swelling continued to evolve even after the NIR light was turned off and the cell volume continued to increase for approximately 5 min. When hypermolar 50 mM dextran was added to the solution, cell swelling was not observed after NIR light exposure as it was not possible for water to flow into cells under this condition (Figure 1Ac, Supplementary Video 3). Thus, after exposing cells previously incubated with the APC to NIR light, ionic pressure gradients across the cellular membrane were impaired due to damage to the plasma membrane that resulted in cell swelling followed by cell bursting. The cell volume change and the 3D cell images show that control conditions did not evoke significant cell damage (Supplementary Figure 1, Supplementary Video 4).

### Water flows into dying cells causing swelling

As summarized in Figure 1Ad, water entered cells immediately after NIR-PIT. Control conditions elicited minimal water exchange between outside and inside of the cell. This result demonstrates that NIR-PIT-induced damage allows water to rapidly flow into cells and is the cause of the observed swelling.

### Intracellular GFP signal dilutes during swelling and is released extracellularly after cell rupture

Dynamic diSPIM imaging and quantitative analysis demonstrated dilution of intracellular GFP (Figure 1Ba, b, Supplementary Video 5, 6) followed by release of GFP after cell membrane rupture (Figure 1Ba, Supplementary Video 5). A small amount of residual GFP was seen in dying cells (Figure 1Ba, Supplementary Video 5). There was no change in control condition (Figure 1Bc, Supplementary Video 7). These findings suggested that NIR-PIT treatment induced rapid membrane rupture after rapid expansion of the cell volume, resulting in release of intracellular contents.

### Fluorescence microscopy with LIVE/DEAD staining demonstrates the permeability of NIR-PIT treated cells to specific macromolecules

When NIR light was applied to Tra-IR700 bound cells, cellular swelling was seen within 30 seconds of NIR exposure (Supplementary Figure 2). Distinct blebs became visible within 3-min after the NIR exposure, but green fluorescent molecules (hydrolyzed Calcein-AM) remained intracellular. Over the next few minutes the green fluorescence gradually disappeared from the cell, and ethidium homodimer (EthD-1) was observed to enter the cell. These results suggest that the cell-membrane damage induced by NIR-PIT initially induces osmotic damage to the membrane permitting the passage of water but not larger or charged molecules. After further cellular membrane rupture, larger molecules can flow into and out of cells.

### NIR-PIT induces ICD and DC maturation

**Figure 2:**
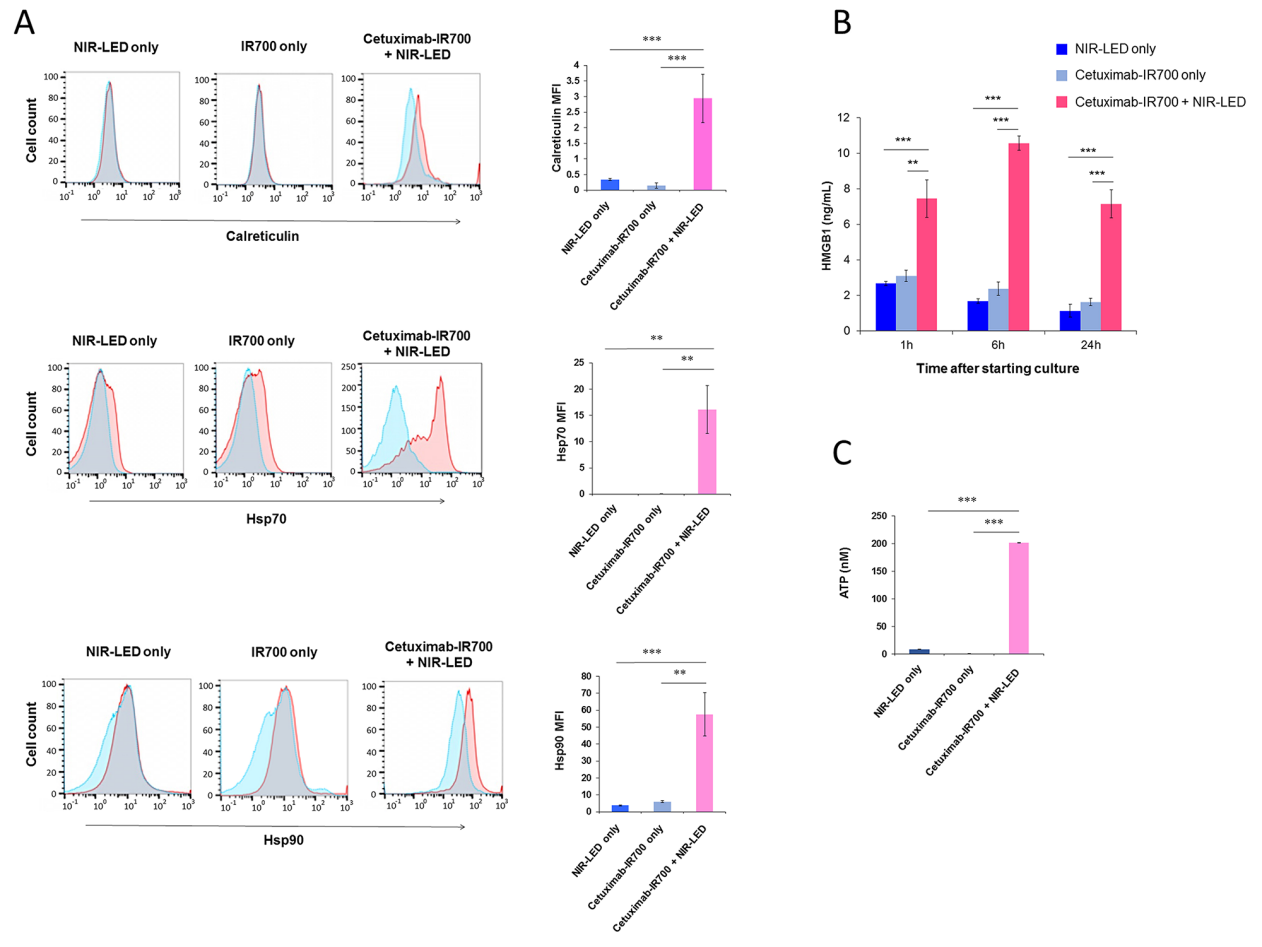
Biological markers of ICD were expressed after NIR-PIT **A**. NIR-PIT induces cell surface exposure of calreticulin, Hsp70, and Hsp90 in A431 cells. Representative flow cytometric histograms showing surface expression of calreticulin, Hsp70, and Hsp90 (red), isotype control (blue) or after mixing with blocking compound (blue), and overlap (gray) in cells exposed to NIR-LED alone (left), cetuximab-IR700 (middle) or NIR-LED plus cetuximab-IR700 (right). Only propidium iodide-negative cells were included in the analysis. Data are representative of 3 to 4 independent experiments. Surface expression levels of calreticulin, Hsp70, and Hsp90 determined by flow cytometry calculated as the median fluorescence intensity (MFI) of cell surface calreticulin, Hsp70, and Hsp90 staining after subtraction of the isotype control MFI. Graphs with error bars indicate mean ± SD from three to four independent experiments. Statistical significance was determined by a one-way ANOVA and Tukey’s post-hoc test. Asterisks denote statistical significance (***P < 0.001). **B**. NIR-PIT induces a rapid release of HMGB1 from cancer cells. A431 cells were incubated with cetuximab-IR700 (10 μg/ml) and exposed to NIR-LED for 20 min. Extracellular HMGB1 release was measured 1, 6, and 24h after NIR-LED exposure. NIR-LED exposure only or incubation with cetuximab-IR700 without NIR-LED exposure (cetuximab-IR700 only) were used as controls. Data are represented as mean ± SD from three replicate wells. Statistical significance was determined by a one-way ANOVA and Tukey’s post-hoc test. Asterisks denote statistical significance (**P < 0.01, ***P < 0.001). **C**. NIR-PIT induces a rapid release of ATP. A431 cells were incubated with cetuximab-IR700 (10 μg/ml) and exposed to NIR-LED for 20 min. Extracellular ATP concentrations were measured. NIR-LED exposure only or incubation with cetuximab-IR700 only were used as controls. Data are represented as mean ± SD from thee replicate wells. Statistical significance was determined by a one-way ANOVA and Tukey’s post-hoc test. Asterisks denote statistical significance (***P < 0.001).

**Figure 3:**
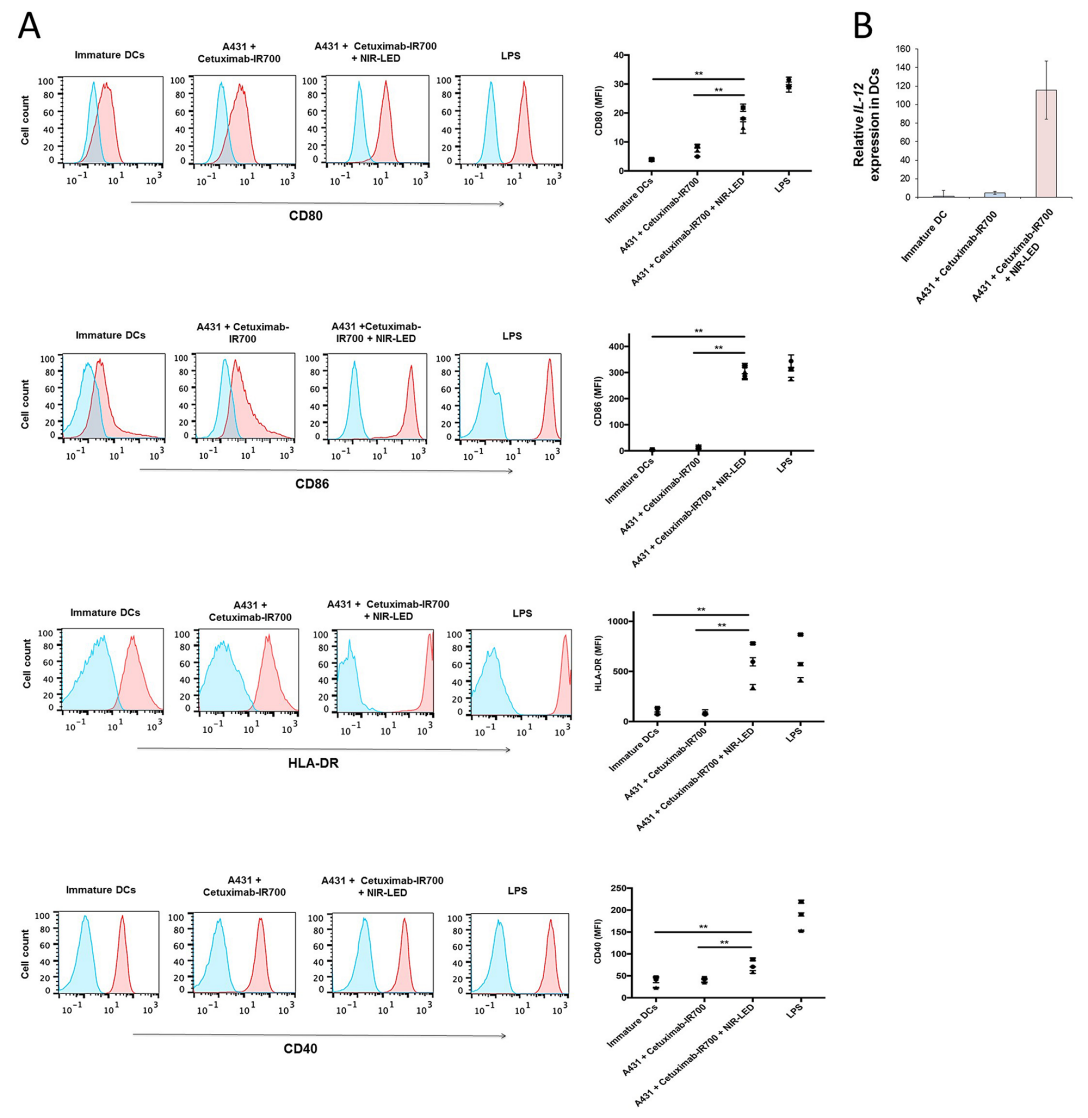
NIR-PIT induced ICD promoted maturation of co-cultured immature dendritic cells **A**. NIR-PIT-treated A431 cells induce DC maturation. Immature DCs (day 5) were cultured with A431 cells treated by NIR-PIT (A431 plus cetuximab-IR700 plus NIR-LED) or cultured with tumor cells incubated with cetuximab-IR700 without NIR-LED exposure (A431 plus cetuximab-IR700). After two-day culture, CD80, CD86, HLA-DR, and CD40 expression on DCs was analyzed by flow cytometry. Immature DCs cultured without stimulation for maturation are shown as a control. DCs stimulated with LPS were used as positive control for DC maturation. Representative flow cytometric histograms showing expression of CD80, CD86, HLA-DR, and CD40 (red) on DCs (left panels). Blue lines indicate staining with isotype control. MFI of CD80, CD86, HLA-DR, and CD40 expression on DCs after subtraction of the isotype control MFI (right panels). Graphs with error bars indicate mean ± SD from biologically independent triplicates from one donor. The graphs represent data from 3 healthy donors (• donor 1; ■ donor 2; ▲donor 3) Statistical significance was determined by two-way ANOVA and Wilcoxon matched-pairs signed rank test. Asterisks denote statistical significance (**P < 0.01). **B**. RT–qPCR analysis of IL-12 gene expression in DCs. NIR-PIT-treated cancer cells induced IL-12 expression in DCs compared with immature DCs or DCs co-cultured with cetuximab-incubated A431 cells. Data are representative of six independent experiments performed using peripheral blood from three healthy donors. IL-12 levels are normalized to the level of expression in immature DCs (cultured DCs without stimulation for maturation). Data are represented as mean ± SD.

ICD relies on the coordinated surface expression and/or release of specific DAMPs. These DAMPs, which include calreticulin, Hsp70, and Hsp90 on the surface of damaged cells in concert with the extracellular release of HMGB1 and ATP provide immunogenic signals to the host immune system that play a crucial role in establishing antitumor immunity and immunological memory [4, 5]. After NIR-PIT, dying cells showed increased plasma membrane expression of calreticulin, Hsp70, and Hsp90 (Figure 2A and Supplementary Figure 3Aa). In addition, NIR-PIT treated tumor cells demonstrated rapid releases of HMGB1 and ATP (Figure 2B, C, Supplementary Figure 3Ab, c). The ability of DCs, which engulf, process, and present antigen from dying tumor cells, to induce successful antitumor immunity relies on their maturation status [5]. Thus we investigated whether NIR-PIT-killed cells induce DC maturation. Co-culture of immature DCs with NIR-PIT-killed tumor cells significantly increased surface expression of the DC maturation markers CD80, CD86, and HLA-DR expression on DCs compared with DCs co-cultured with Cet-IR700-coated tumor cells without NIR-LED exposure or DCs cultured without stimulation for maturation (immature DCs), indicating NIR-PIT-killed cells induced DC maturation (Figure 3A and Supplementary Figure 3Ba). DCs co-cultured with NIR-PIT-killed tumor cells also induced a small but significant increase in expression of the DC costimulatory receptor CD40 expression compared with immature DCs. We investigated interleukin-12 (IL-12) expression in DCs. IL-12 is a cytokine that drives T helper type 1 (Th1) differentiation and play a critical role in immune response against tumor [17, 18]. NIR-PIT-killed tumor cells also increased IL-12 production in DCs (Figure 3B and Supplementary Figure 3Bb). These results suggest that NIR-PIT triggers ICD and has the potential to mediate optimal antitumor immunity.

## DISCUSSION

Based on conventional 2D fluorescence microscopy, it is known that NIR-PIT induces treated cells to swell, and form blebs before dying due to cell rupture [7, 19]. LC-QPM can visualize dynamic changes within live 3D cells by focusing on the strong scattering of the plasma membrane lipid bilayer with high spatial and temporal resolution without staining. LC-QPM demonstrated that the cells expanded rapidly, and then burst dramatically in response to NIR-PIT (Figure 1Aa, Supplementary Video 1). When cells were exposed to only 5 seconds of NIR light, the rate of cell swelling was reduced but continued even after excitation light was switched off (Figure 1Ab, Supplementary Video 2). Another novel technology, diSPIM, can visualize the dynamic three-dimensional behavior of fluorescent proteins in live cells, by exposing light only to a selective plane of interest and sweeping this excitation plane through the volume to build a 3D image stack. Therefore, unlike conventional fluorescence microscopy, photo-bleaching with diSPIM is greatly reduced permitting long sequential observation of a single cell. These observations with novel microscopies, combined with prior experience with confocal microscopy suggest that NIR-PIT induces a rapid alteration in cell volume, which continues even after NIR light is stopped. The cause of the cell swelling can be inferred from several experiments. GFP within the cytoplasm did not decrease but was rapidly diluted after NIR-PIT, and dramatically decreased after cell rupture consistent with the rapid influx of water into the cell, followed by cell bursting and dispersion of cellular contents. The plasma cell membrane is normally a barrier that maintains the osmotic gradient between the outside and inside of the cell creating a membrane pressure. Membrane pressure is a result of surface tension on the lipid bilayer and the cytoskeleton, which supports the cell membrane mostly on the inner surface of the cell. NIR-PIT-induced damage to the plasma membrane impairs the membrane function as a pressurized barrier resulting in the influx of water along the ionic pressure gradient between the inside and outside of the plasma cell membrane. Because NIR-PIT has been observed to occur at 4°C, it occurs even when other biologic processes are largely stalled [7]. Thus, we hypothesized that NIR-PIT induced cell membrane damage by a photo-mediated physico-chemical reaction leads to swelling and bursting of cells (Supplementary Video 1, 2). This was confirmed by repeating QPM experiments with highly osmotic media (50 mM dextran). When the osmotic pressure of extracellular fluid was increased, the cell expansion was inhibited, a result of decreased water in-flow (Figure 1Ac, Supplementary Video 3). The molecular mechanism of causing cell membrane disruption is currently under investigation mostly using analytical chemistry technologies including mass-spectroscopy for detecting changes on membrane lipid and APC and atomic force microscopy for conformational changes on antibody and target proteins on the cellular membrane.

By using small fluorescent dyes, we observed that the initial increase in permeability was limited to water. Influx of radiolabeled water but not larger molecules, was increased during the initial cell expansion immediately after NIR-PIT-treatment (Figure 1Ad). However, neither hydrolyzed Calcein AM (MW 622.55), nor a complex of EthD-1 and nucleic acids went across the cellular membrane early in the process. Later, after the cell membrane ruptured, these larger molecules were able to enter and leave the cells (Supplementary Figure 2B). This observation is consistent with the cytoplasmic GFP we visualized with diSPIM, which dramatically decreased after cell rupture (Figure 1Ba and Supplementary Video 5).

Biological cell death, either occurring naturally or as a result of drug therapy, largely relies on apoptotic or programmed cell death [20, 21]. Apoptotic cell death is an orderly multistep process that takes hours culminating in cell death [22]. During the process of apoptosis, cells gradually shrink in size but the plasma membrane remains largely intact. In contrast, physical or chemical stress such as occurs in NIR-PIT, results in rapid necrotic cell death, typically involving rupture of the plasma membrane and dispersal of cellular contents into the microenvironment. During necrotic cell death, bioactive molecules such as DAMPs and tumor-antigens may be released and stimulate antigen-presenting cells to mount a long-lasting immune response against tumor [23]. The antitumor immunity elicited by an ICD inducer may subsequently kill tumor cells that survive the original damage at the treatment site and tumor cells that are distant from the treatment site [6, 24].

The discovery of NIR-PIT as a new ICD inducer and the understanding of underlying mechanisms may facilitate the integration of NIR-PIT and cancer immunotherapy [6, 24]. ICD cannot be achieved by cancer cell death that is not accompanied by calreticulin exposure, ATP and HMGB1 release. The absence of one of the signature components of ICD has been reported to result in failure to elicit adaptive immune responses against tumor [4, 6]. We previously demonstrated a rapid release of ATP *in vivo* as assessed by bioluminescence imaging, when tumors are treated with NIR-PIT [8]. In the present study, we demonstrated the cardinal features of ICD; exposure of surface calreticulin, Hsp70, Hsp90 and release of ATP and HMGB1 (Figure 2 and Supplementary Figure 3A), which also induce significant DC maturation and increased expression of the *IL-12* gene in DCs (Figure 3 and Supplementary Figure 3B) [4, 5, 25]. These results suggest that NIR-PIT triggers ICD and may generate long-lasting antitumor immunity in cancer patients. Additionally, we have tried to examine DC maturation after PIT *in vivo* in a mouse A431 tumor model. CD86 positive population of DC increased in CD11c, CD205, MHC-class II positive cells that indicated DC maturation was induced (Supplementary Figure 5). However, the difference was not significant in other maturation markers such as CD80 and CD40 probably because implanted tumor cells induced DC maturation. Therefore, we could hardly see partial difference of further increased maturation marker of DC after NIR-PIT. We can overcome this issue by using an immunocompetent mouse model with a syngeneic mouse tumor and an antibody recognizing a murine target antigen expressed on the tumor cells. However, unfortunately, such model is not currently available. Now we are establishing syngeneic mouse tumor models that can be targeted with an antibody. As soon as we will establish a model, we will investigate anti-tumor immunity induced with NIR-PIT in the syngeneic mouse model. Orthotopic dual-color model should be helpful to investigate the tumor-host interaction [26–29]. Contribution of cytotoxic T-cells could be readily investigated with the mouse model.

While the concept of targeted light therapy with photosensitizers, commonly known as photodynamic therapy (PDT), was established over three decades ago, [30, 31] most PDT photosensitizers permeate into cells and after exposure to light, cause apoptotic cell death, and do not target tumors with sufficient selectivity or efficiency to induce ICD or avoid damage to adjacent normal cells [32–34]. Attempts to more precisely target PDT photosensitizers have been limited by the hydrophobicity of the photosensitizer, which dominates the pharmacokinetics *in vivo* despite conjugation to targeting antibodies. Antibody photosensitizers based on PDT agents typically accumulate in the liver due to the overall hydrophobicity of the APC. However, if the photosensitizer is highly hydrophilic as is the case with IR700, then the pharmacokinetics of the APC are dominated by the antibody and *in vivo* targeting is dictated by the presence of antigen on the target cell. Therefore, APCs using IR700 successfully bind cancer specific cell surface molecules after intravenous injection and efficiently kill target cancer cells only upon NIR light exposure with immunogenic cell death.

**Figure 4:**
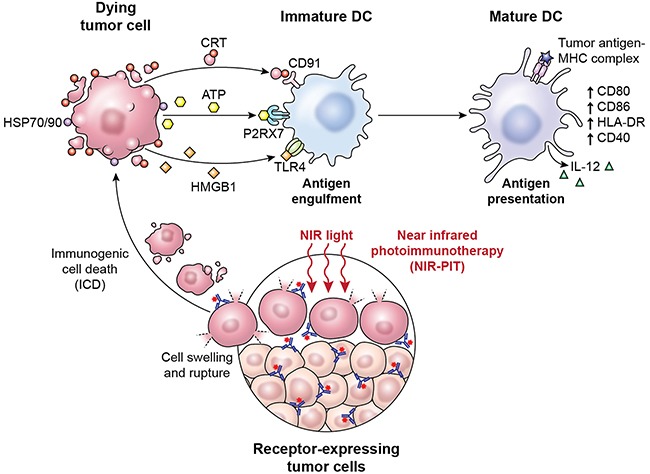
A scheme explaining the mechanism of NIR-PIT induced ICD

In conclusion, these results indicate that NIR-PIT induces alterations in the cell membrane leading to equilibration of osmotic pressures between the inside and outside of the cell and water flowing into the cell. When the cell volume increases to a critical point, the cell membrane forms blebs and ruptures, thus becoming highly permeable to larger molecules. The release of DAMPs from the cytosol and nucleus triggered by NIR-PIT induces DC maturation (Figure 4). This series of events has been shown to be associated with adaptive immunity, induction of immunologic memory, and promotion of an effective host antitumor immune response, including an abscopal effect at sites distal from NIR-PIT therapy. We are currently testing the impact of NIR-PIT on systemic immunity, including surrogates of ICD, in the first-in-human NIR-PIT clinical trial (NCT02422979). The predictive and pharmacodynamic biomarker data from this trial may assist in optimization of NIR-PIT as a cancer treatment modality for localized disease, and potentially as a component of a multimodality regimen incorporating systemic immunomodulatory therapy.

## MATERIALS AND METHODS

### Reagents

NHS ester of IR700 was purchased from LI-COR Bioscience (Lincoln, NE, USA). Trastuzumab, a humanized anti-HER-2 MoAb was purchased from Chugai Pharmaceutical Co., Ltd. (Tokyo, Japan). Cetuximab, a humanized anti-EGFR MoAb was purchased from Bristol-Meyers Squibb Co (Princeton, NJ, USA). All other chemicals used were of reagent grade.

### Preparation of IR700 conjugated antibodies

Trastuzumab or cetuximab (1 mg, 6.8 nmol) was incubated with the NHS ester of IR700 (34.2 nmol,) in 0.1M Na_2_HPO_4_ (pH 8.5) at room temperature for 30 min. Then, the reaction mixture was applied to a gel-filtration column (Sephadex G50, PD-10, GE Healthcare Milwaukee, WI, USA), and the derived trastuzumab-IR700 or cetuximab-IR700 conjugate (Tra-IR700 or Cet-IR700) was eluted with PBS. The recovery of antibody was 95%. The concentration of IR700 was measured by absorption with spectroscopy to determine the number of IR700 molecules conjugated to each antibody. The number of IR700 molecules per antibody molecule was approximately 3.

### Cell preparation

HER2-gene-transfected NIH3T3 (ATCC, Manassas, VA) cells (3T3-HER2) were used. The cells were cultured in a glass–bottom culture well in RPMI 1640 containing 10% fetal bovine serum (Invitrogen, Carlsbad, CA, USA), 0.03% L-glutamine, 100 units/mL penicillin, and 100 μg/mL streptomycin in 5% CO_2_ at 37°C.

### Animal and tumor models

All *in vivo* procedures were conducted in compliance with the Guide for the Care and Use of Laboratory Animal Resources (1996), US National Research Council, and approved by the National Cancer Institute Animal Care and Use Committee. Six- to eight-week-old female homozygote athymic nude mice were purchased from Charles River (NCI-Frederick, Frederick, MD). A431 cell line (2 × 10^6^, ATCC) was injected subcutaneously in the dorsa of the mice under isoflurane anesthesia. Tumors were studied after they reached volumes of approximately 100 mm^3^. NIR-PIT was performed with 100 μg of Cet-IR700 *i.v*. via the tail vein, NIR light was exposed to tumors at 100 J/cm^2^ on day 1 after Cet-IR700 *i.v*. injection as previously described^7^.

### Optical setup of 3D LC-QPM

The details of 3D LC-QPM setup were published by T. Yamauchi et.al [13, 14]. Light emitted from a halogen lamp (center wavelength λ_c_ = 800 nm) passed through a Michelson interferometer equipped with two identical water-dipping objective lenses (Nikon CFI Fluor 60xW; Water Immersion, NA = 1.0). The reflected wavefronts from the sample and the reference mirror were focused onto the CCD camera so that they form interference images. Based on the principle of low-coherence interference (also known as “white-light interference”), the interference images can only be observed when the optical-path-length from the beamsplitter to the sample and the one to the reference mirror are precisely balanced. The vertical sectioning depth of the low-coherence interference, which is defined by the optical coherence length, is inversely proportional to the spectral bandwidth of the light source and was 0.93 μm in our setup, while the lateral resolution of the microscope calculated as a diffraction limit was 0.49 μm.

During the image acquisition, we vertically scanned the sample up and down with the piezoelectric transducer (PZT2) beneath the sample. By low-coherence interference, we can assume a virtual plane where a horizontal cross section of the sample is obtained. The positions of the beam splitter, objective lenses, and reference mirror are fixed and only the sample moves up and down. By sequentially moving the sample across the sectioning plane and we can obtain a z-stack of horizontal cross sections. In the vicinity of the sectioning plane, we can see the oscillation of the interference fringes and the period of the oscillation is 328 nanometers of the z-scan, which is half of the wavelength of the light source in the culture medium. In order to extract the amplitude of the oscillation, we obtained interference images by vertically stepping 82 nanometers at a time, which is one quarter of the oscillation period. From four consecutive interference images; *I*_1_, *I*_2_, *I*_3_, and *I*_4_, the amplitude of the oscillation is extracted as Ampl = (*I*_1_-*I*_3_)^2^ + (*I*_3_ -*I*_4_)^2^. Because the modulation amplitude of the interference image is proportional to the reflectivity of the objects in the vicinity of the sectioning plane, we can visualize the 3D morphology of the cells by rendering the z-stack of the modulation amplitude images. We retrieved 50 slices of the modulation amplitude images to render a 3D dataset, whose z-scanning depth was 16.4 μm. It took 12 seconds to obtain the 3D dataset, from which the cell volume was calculated.

The output light from the halogen lamp is spectrally filtered by a long-pass filter (LPF) and a dichroic mirror (DM). The DM, which reflects the light from 400 nm to 950 nm, is always in position, whereas the LPF which filters light shorter than 780 nm can be manually displaced from the optical path when we expose the cells to NIR light for therapy. Because IR700 does not absorb light at wavelengths longer than 780 nm, the cells remain intact as long as the LPF is in front of the light source.

### Observation by 3D LC-QPM

Tra-IR700 (10 μg/mL, 0.1 μM as IR700) was added to the culture medium, and 3T3-HER2 cells were incubated for 24 hr, washed with PBS, and observed with 3D LC-QPM in 2 mL of RPMI 1640. To increase osmotic pressure, 50 mM dextran (MW∼6,000) was added to the medium. The baseline data were obtained with a long-pass filter, which eliminates wavelengths less than 780 nm, before each observation period. For continuous irradiation studies, NIR light (700-950 nm) was irradiated continuously throughout the observation duration. For 5-sec irradiation studies, the cell was exposed to NIR light (700-950 nm) for 5 sec, and then the observation was continued with the long-pass filter.

### LIVE/DEAD staining

For assessment of cell viability the LIVE/DEAD Viability/Cytotoxicity Kit (Molecular Probes, Eugene, OR, USA) was used. Calcein AM can permeate the cell plasma membrane, and is retained in live cells after hydrolysis (MW 622.55 after hydrolysis) (Supplementary Figure 2A). Ethidium homodimer (EthD-1, MW 856.77) cannot penetrate into live cells due to its strong positive charge, but can penetrate into the membranes of dead cells with damaged plasma membranes where it binds to nucleic acids. 3T3-HER2 cells were cultured in a glass–bottom culture well and incubated with Tra-IR700 (10 μg/mL) for 24hr. The well was set-up on the fluorescence microscope (Olympus IX73 microscope, Olympus Corporation, Tokyo, Japan) and LIVE/DEAD reagents were added to the well. Then the cells were exposed to NIR light for 30 sec using the Cy7 filter set (excitation: 658-758 nm) and sequential images were obtained for 10 min after the light exposure.

### Water flow-in assay

To assess the changes in water permeability during NIR-PIT, 3T3-HER2 cells were incubated in radiolabeled water ([^3^H]H_2_O) added to the culture medium. The cells were cultured in a plastic-bottom culture well (f30 mm), and incubated with Tra-IR700 (10 μg/mL) for 24 hr (n=6). Then, [^3^H]H_2_O was added to the medium and cells were irradiated with a NIR light-emitting diode (LED) (670-710 nm, L690D-66-16100, Epitex Inc., Kyoto, Japan) for 10 min (2 J/cm^2^). For a control, non-irradiated and/or samples not exposed to Tra-IR700 were prepared (n=6). After the irradiation, the incubation medium was removed and the cells were collected after trypsinization. The radioactivity in the medium and cells was measured with a liquid scintillation counter (LSC-6100, Hitachi Aloka Medical, Ltd., Tokyo, Japan).

### Dual-view inverted selective plane illumination microscopy (diSPIM)

DiSPIM enables fast volumetric imaging with minimal photo-bleaching and photodamage [15]. DiSPIM uses two perpendicular objectives (40x, 0.8 NA water immersion lenses) to alternately excite the sample with a sheet of light and detect planar fluorescence. By switching the excitation and detection between objectives in an alternating duty cycle, two orthogonal, volumetric sample views are acquired for each time point. Isotropic resolution is achieved by fusing the two views using joint-deconvolution.

The sample (5 × 10^5^ 3T3-HER2-GFP cells) was prepared on a rectangular coverslip (24×50 mm, # 1.5, VWR International, Cat. # 48393-241) that was placed in a sample chamber (Applied Scientific Instrumentation, Eugene, OR, USA, Part # I2450) and mounted in a fiber-coupled diSPIM [16]. To image cells, the sample was translated with a motorized stage until it was positioned at the focus of the two objectives (40x, NA 0.8, Nikon, Cat. # MRD07420). A 488 nm laser was used for excitation and a 488 nm long pass filter (Cat. # LP02-488, Semrock) was placed in each emission arm to block excitation. Scientific CMOS (Orca Flash 4.0 V2 from Hamamatsu) cameras were used for detection. Each volumetric view consisted of 40 planes (planes were spaced 1 μm apart and collected every 5 ms, with 2.5 ms exposure time per plane). A background image stack was acquired by turning off the laser, and the average background intensity was subtracted from each image stack before registration and joint deconvolution using an open source diSPIM plug-in in the MIPAV (Medical Image Processing, Analysis, & Visualization) programming environment [16]. Dual-view volumes were acquired every 5 seconds for 30 minutes. To test the effect of NIR-PIT, the cells were irradiated with an NIR laser (for 60 seconds, 500 mW) during diSPIM image acquisition.

### Image analysis of diSPIM

Fluorescence signal intensity of cells on diSPIM images (two single views data registered and deconvolved) was unsteady compared to that on single view iSPIM images. Therefore, we used single view iSPIM images for image analysis.

All images were analyzed using Image J software (http://rsb.info.nih.gov/ij/). Cells and background was chosen as the regions of interest (ROIs). First, we measured signal intensity (SI) of cells and calculated relative intensity (RI) of each cell using the following equation: RI = current signal intensity / initial signal intensity. Next, contrast-to-noise ratio (CNR) was calculated using the following equation: CNR = (average SI of cell - that of background) / standard deviation of SI in cell. We defined cell on images if SI is in the value between CNR ± CNR/2 and calculated the volume of cells. Finally, relative GFP, expressing intracellular GFP amount, was calculated using the equation: relative GFP = RI x volume of each cell.

### Detection of cell surface ICD markers

HER1-expressing human epidermoid carcinoma cells (A431, ATCC) and human breast cancer cells (MDA-MD-468, ATCC) were used. Tumor cells were incubated with Cet-IR700 (10 μg/mL) for 15 min at 4°C, washed, re-suspended in RPMI 1640 medium (Invitrogen) supplemented with 10% fetal bovine serum (Invitrogen) and exposed to a NIR-LED (690 ± 20 nm, 0.4 A) for 15 min. After exposure to NIR irradiation, the cell surface expression levels of calreticulin (Alexa Fluor 488-conjugated, clone 326203, R&D Systems, Minneapolis, MN, USA) and Hsp70 (PE-conjugated, clone C92FaA-5, Enzo Life Sciences, Farmington, NY, USA) were analyzed by flow cytometry using specific monoclonal antibodies and appropriate isotype controls. To detect cell surface Hsp90 expression, HS-27 (FITC-conjugated tethered Hsp90 inhibitor, 5 μM) was used [35]. To block the binding of HS-27 and evaluate cell surface Hsp90-specific binding, HS-10 (parent Hsp90 inhibitor, 200 uM) was added before adding HS-27. Hsp90-specific median fluorescence intensity (MFI) was calculated as the HS-27 MFI after subtraction of the MFI in the surface Hsp90-blocking condition (HS-10 + HS-27). Permeabilized cells were detected by propidium iodide (PI; BioLegend, San Diego, CA, USA) or H33258 (Invitrogen, Carlsbad, CA, USA), and removed from the analysis as described previously [4]. Flow cytometric analyses were performed using a MACSQuant Analyzer (Miltenyi Biotec, Bergisch Gladbach, DE, Germany) as described previously [36–38]. Data were analyzed using FlowJo software (FlowJo LLC., Ashland, OR, USA).

### HMGB1 ELISA

Tumor cells were incubated with Cet-IR700 (10 μg/mL) for 15 min at 4°C and washed. Cells were resuspended with RPMI 1640 medium supplemented with 10% fetal bovine serum and exposed to a NIR-LED (690 ± 20 nm, 0.4 A) for 20 min. The cells were seeded at a density of 5 × 10^5^ cells per 35-mm dish with 2.5 mL of medium and incubated at 37°C in 5% CO_2_. After incubation for the indicated amount of time (1, 6 and 24 h), the culture supernatants were collected and centrifuged, and the concentration of HMGB1 in the supernatant was measured using an ELISA kit (IBL International, Hamburg, Germany) according to the manufacturer’s instructions. Tumor cells exposed to NIR-LED irradiation without incubation with Cet-IR700, or cells incubated with Cet-IR700 without NIR-LED exposure were used as controls.

### ATP assay

Tumor cells were incubated with Cet-IR700 (10 μg/mL) for 15 min at 4°C and washed. Cells were suspended with medium (1 × 10^6^ cell/mL) and exposed to a NIR-LED (690 ± 20 nm, 0.4 A) for 20 min. Extracellular ATP concentrations in the culture supernatants were measured by a luciferin-based ATP Assay (ENLITEN, Promega, Madison, WI, USA) [4, 39]. Tumor cells exposed to NIR-LED irradiation without incubation with Cet-IR700 or cells incubated with Cet-IR700 without NIR-LED exposure were used as controls.

### Flow cytometric analysis of dendritic cell phenotype after interaction with NIR-PIT-exposed tumor cells

Peripheral blood mononuclear cells (PBMCs) were isolated by Ficoll (GE Healthcare Bio-Sciences, Pittsburgh, PA) gradient centrifugation of buffy coats obtained from healthy donors by the National Institutes of Health Department of Transfusion Medicine on an IRB-approved protocol. CD14+ cells were isolated from PBMCs by positive selection on a MACS column using CD14 microbeads (Miltenyi Biotec). Dendritic cells (DCs) were generated by culture in the presence of 50 ng/ml granulocyte-macrophage colony-stimulating factor (GM-CSF) (PeproTech, Rocky Hill, NJ, USA) and 10 ng/mL interleukin-4 (IL-4) (PeproTech) as described [25]. Tumor cells were incubated with Cet-IR700 without NIR exposure or exposed to NIR-PIT with Cet-IR700 and co-cultured for 24-48 hours with immature DCs (day 5) at a DC/tumor cell ratio of 1:1. DCs stimulated with 100 ng/mL of lipopolysaccharide (LPS) (Sigma, St. Louis, MO, USA) for 12 hours were used as a positive control for DC maturation. CD80 (Alexa Fluor® 488-CD80 clone 2D10), CD86 (PE-CD86 clone IT2.2), CD40 (Pacific Blue-CD40 clone 5C3) and HLA-DR (PE/Cy7-HLA-DR clone L243) expression on DCs was monitored by flow cytometry. The details of gating strategy are shown in Supplementary Figure 4. DCs were stained with specific monoclonal antibodies (mAb, all from BioLegend, San Diego, CA, USA) for 20 minutes at 4°C, washed and analyzed by flow cytometry. Live cells were discriminated by means of LIVE/DEAD Fixable Aqua Dead Cell Stain (Life Technologies, Grand Island, New York, USA) and dead cells were excluded from all analyses.

### Quantitative reverse-transcription polymerase chain reaction (RT-qPCR)

Total RNA was isolated from DCs using the RNeasy RNA Isolation Kit (Qiagen). The RNA was reverse transcribed (Applied Biosystems, Foster City, CA, USA) and IL-10 mRNA was detected by RT-qPCR using the ABI 7500 (Life Technologies, Grand Island, NY, USA) as described previously [40]. RT-qPCR experiments were performed in triplicate. The primer sequences for RT-qPCR were as follows: IL-12, forward 5′-CATCTCTTGGTTTTCCCTGGTT-3′ and reverse 5′-CAATCCAATTCTACGACATAAACATCTT-3′ 18S rRNA, 5′-AGTCCCTGCCCTTTGTACACA-3′ and 5′-CGATCCGAGGGCCTCACTA-3′.

### Statistics

All results were reported as means with standard deviation (SD). For multiple comparisons, a one-way analysis of variance (ANOVA) with a Tukey’s multiple comparisons test was used. For the analysis of diSPIM data, Dunnett’s multiple comparisons test was used. *P* values of less than 0.05 were considered significant. The analyses were performed with GraphPad Prism software version 6.0 (GraphPad Software Inc., La Jolla, CA, USA)

## SUPPLEMENTARY FIGURES AND VIDEOS
















