# Supplementary material for: Immunogenic cancer cell death selectively induced by near infrared photoimmunotherapy initiates host tumor immunity
Source: Oncotarget. 2017 Jan 2;8(6):10425–36. doi: 10.18632/oncotarget.14425 (PMC5354669; doi:10.18632/oncotarget.14425)
Supplement: Supplementary file 1 [file oncotarget-08-10425-s001.pdf]

Immunogenic cancer cell death selectively induced by near infrared photoimmunotherapy initiates host tumor immunity

SUPPLEMENTARY FIGURES AND VIDEOS

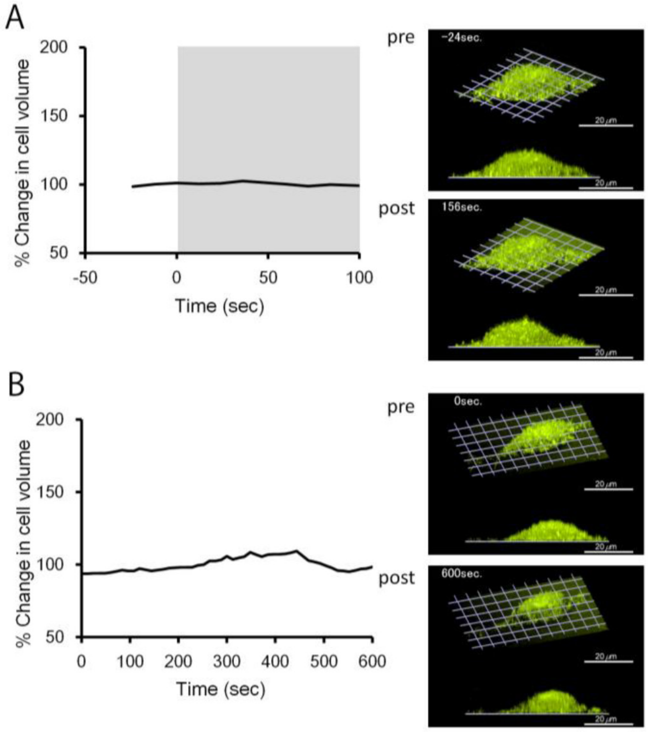

Supplementary Figure 1: 3D LC-QPM imaging in control conditions. 3D LC-QPM observation in continuous light exposure without Tra-IR700 conjugate **A.** and no NIR light exposure with Tra-IR700 conjugate **B.** The cell volumes were not changed in these conditions. The gray area in graph A represents the NIR light exposure duration.

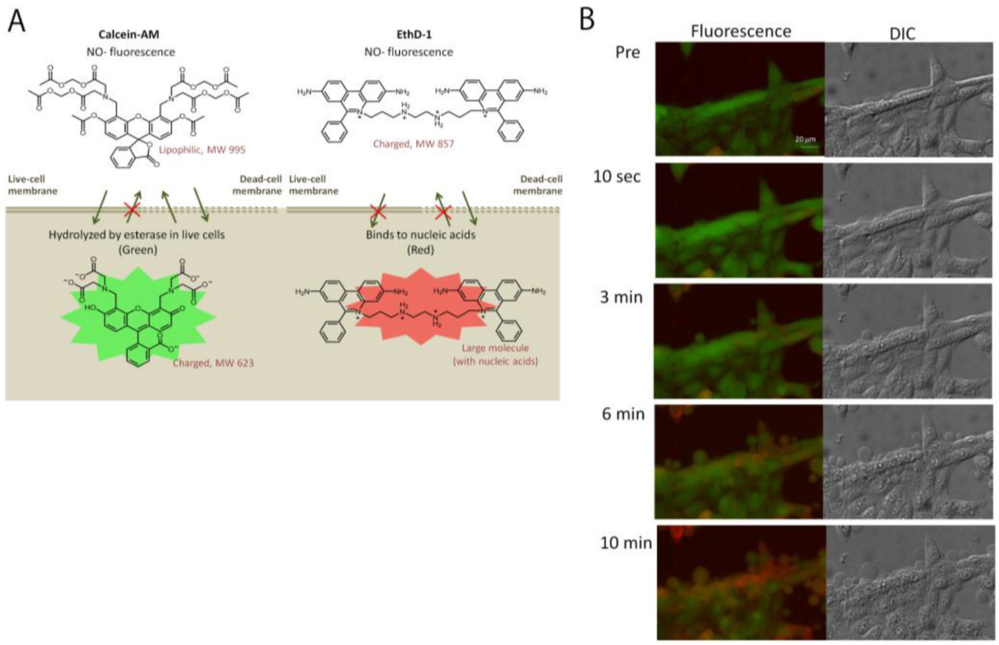

Supplementary Figure 2: LIVE/DEAD staining during NIR-PIT. A. In LIVE/DEAD staining, the live cells are stained in green with Calcein-AM derivative, and the dead cells are stained in red with EthD-1 nucleic acid complex. Calcein-AM penetrates the cell plasma membrane with its lipophilicity and hydrolyzed by esterase in live cells. The hydrolyzed molecule (MW 623) has green fluorescence and is retained in the cell due to its hydrophilicity in live cells, but it is leaked out as a consequence of plasma membrane destruction. On the other hand, EthD-1 (MW 857) cannot penetrate the live cell membrane due to its charge, but it can go into the dead cell after cell membrane damage. Then it binds to nucleic acids and emits red fluorescence. **B.** Fluorescence and DIC images before and after NIR exposure. Blebs were seen from 3-min after the NIR exposure, but green fluorescence remained within the cells. Gradually, the green fluorescence disappeared from the cell, and then EthD-1 was observed to enter cells after severe damage.

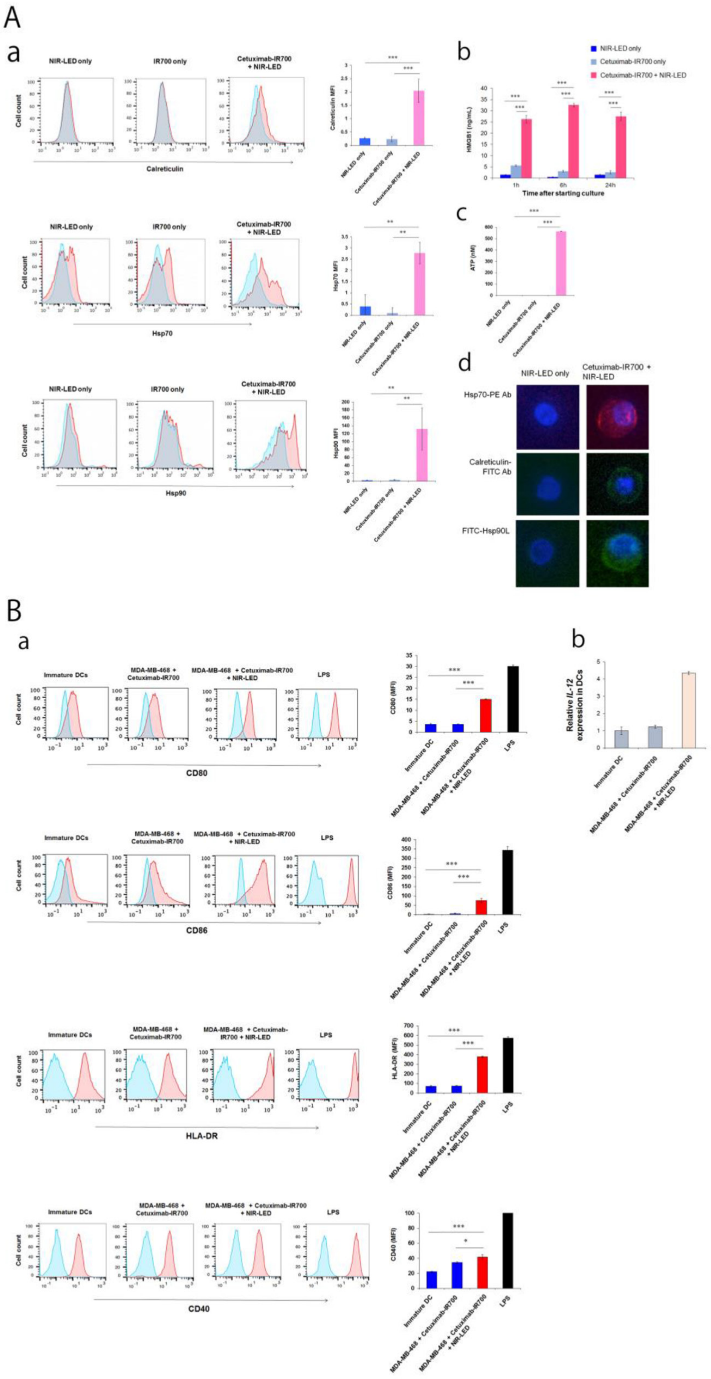

Supplementary Figure 3: A. (a) Representative flow cytometric histograms in MDA-MB-468 cells showing surface expression of calreticulin, Hsp70, and Hsp90 (red), isotype control (blue) or mixing with blocking compound (blue), and overlap (gray) in cells exposed to NIR-LED alone (left), cetuximab-IR700 (middle) or NIR-LED plus cetuximab-IR700 (right). ***$P < 0.001$. (**b**) NIR-PIT induces a rapid release of HMGB1 from cancer cells (MDA-MB-468). Data represent the mean ± SD from three replicate wells. **$P < 0.01$, ***$P < 0.001$. (**c**) NIR-PIT induces a rapid release of ATP from MDA-MB-468 cells. (**d**) Fluorescence microscopy showed expression of calreticulin, Hsp70, and Hsp90 on the cell surface. **B.** (**a**) NIR-PIT-treated MDA-MB-468 cells increase CD80, CD86, HLA-DR, and CD40 expression on DCs. Representative flow cytometric histograms showing expression of CD80, CD86, HLA-DR, and CD40 (red) on DCs (left panels). Blue lines indicate staining with isotype control. MFI of CD80, CD86, HLA-DR, and CD40 expression on DCs after subtraction of the isotype control MFI (right panels). ***$P < 0.001$. (**b**) RT–qPCR analysis showed the induction of IL-12 gene expression in DCs exposed to NIR-PIT-treated MDA-MB-468 cells. IL-12 levels are normalized to the level of expression in immature DCs (cultured DCs without stimulation for maturation). Data are represented as mean ± SD.

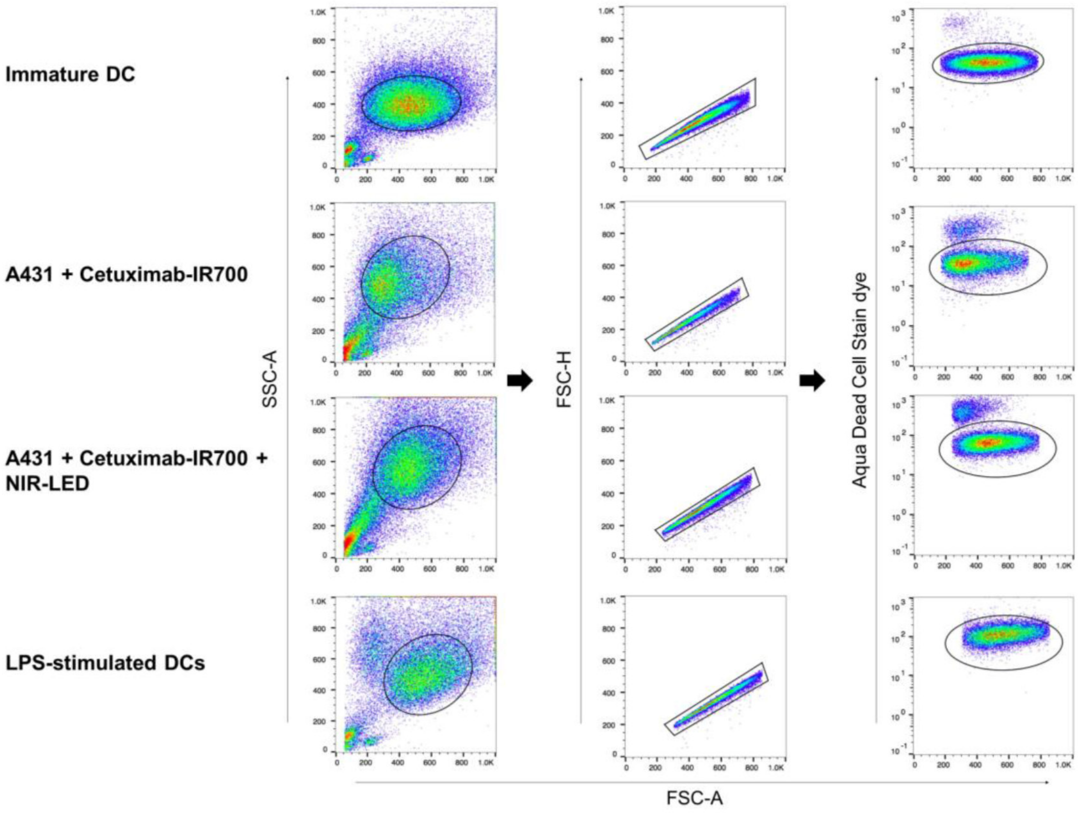

Supplementary Figure 4: Gating strategy for DCs. Tumor cells were incubated with Cet-IR700 without NIR exposure or exposed to NIR-PIT with Cet-IR700 and co-cultured for 48 hours with immature DCs (day 5) at a DC/tumor cell ratio of 1:1. Floating cells were collected and stained with CD80, CD86, HLA-DR, and CD40. The floating immature DCs and DCs stimulated with LPS for 12 hours were also collected and stained with these antibodies and used as negative and positive controls respectively. DCs were defined by size and granularity (left column; FSC-A vs SSC-A), single cells (middle column; FSC-A vs FSC-H), and live cells (right column; Aqua Dead Cell Stain dye-negative cells). The size and granularity of DCs co-cultured with NIR-PIT-killed cancer cells was similar to LPS-induced mature DCs.

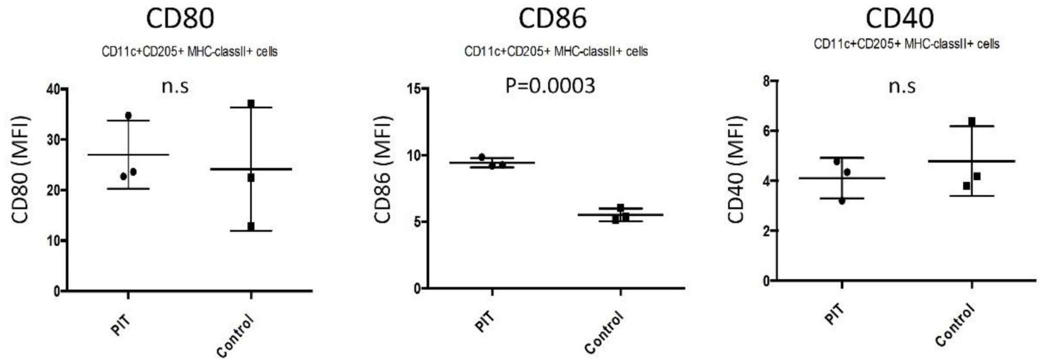

Supplementary Figure 5: *In vivo* study in tumor bearing nude mice. A431 cells were injected subcutaneously in the dorsum of the nude mice (n = 3). 10 days after the cell injection, cetuximab-IR700 (100 µg) was injected intravenously, and the tumors were exposed to NIR-LED (50 J/cm^2) for PIT. For control, the mice were not expose to NIR-LED (n = 3). The cells were collected from NIR-PIT treated and non-treated tumors, and CD80, CD 86 and CD 40 expression in CD11c, CD205, MHC-class II positive cells were monitored by flow cytometry. CD86 was significantly elevated by PIT (P = 0.0003), however, difference was not significant in other maturation markers, CD80 and CD40.

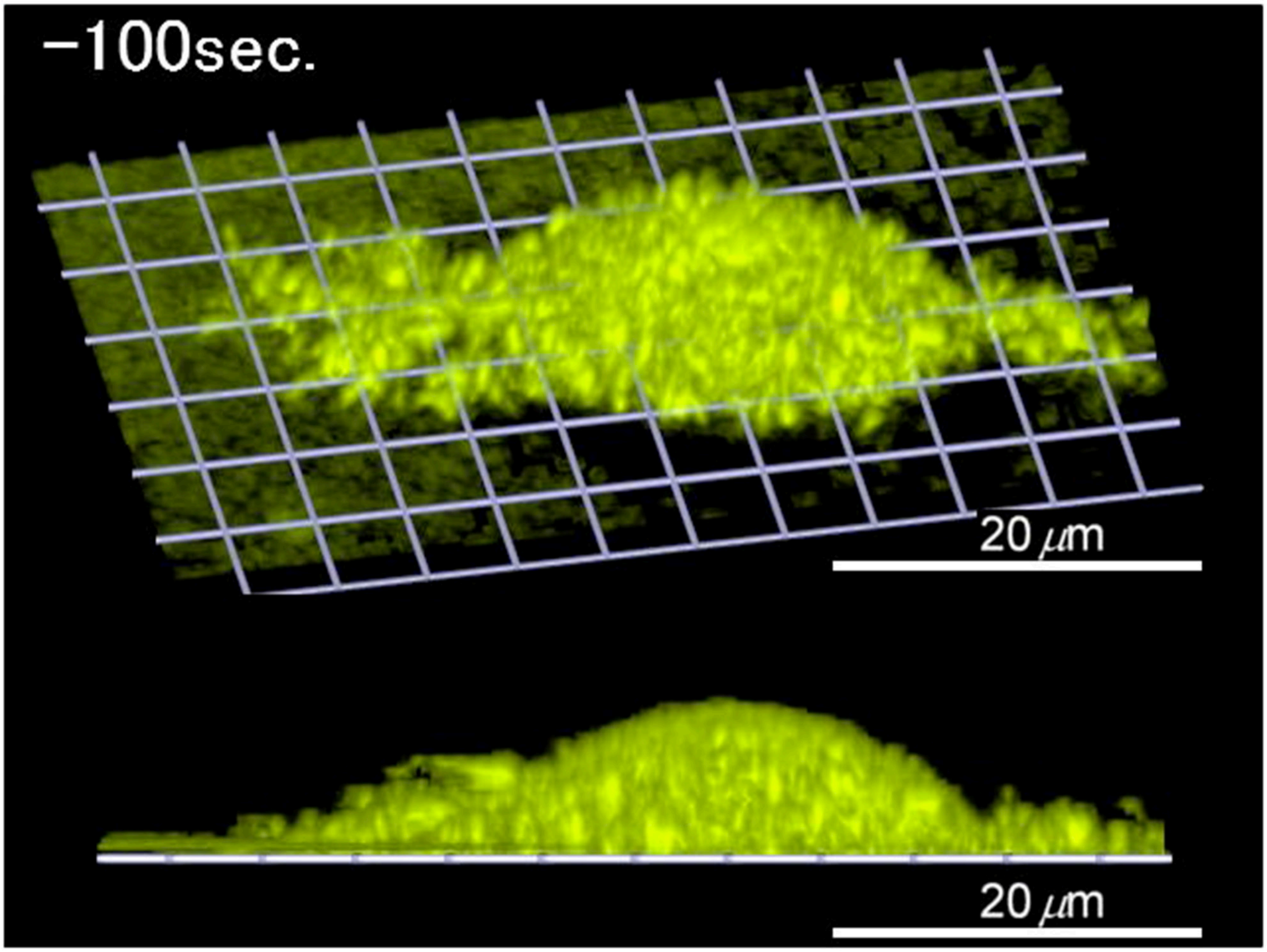

Supplementary Video 1: Volumetric movie in 3D LC-QPM with continuous NIR light exposure in Tra-IR700 treated 3T3-HER2 cell.

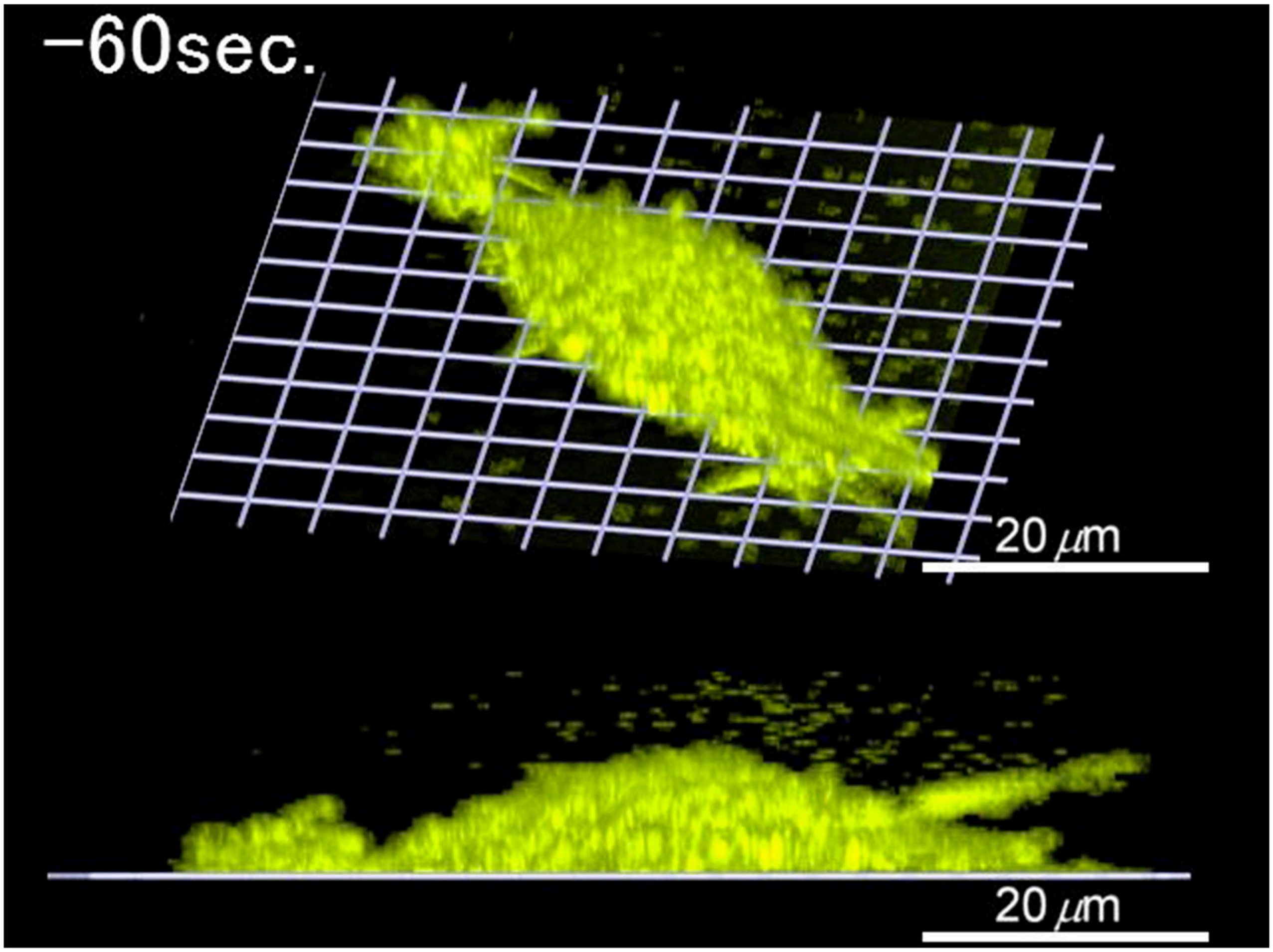

Supplementary Video 2: Volumetric movie in 3D LC-QPM with 5-sec NIR light exposure in Tra-IR700 treated 3T3-HER2 cell.

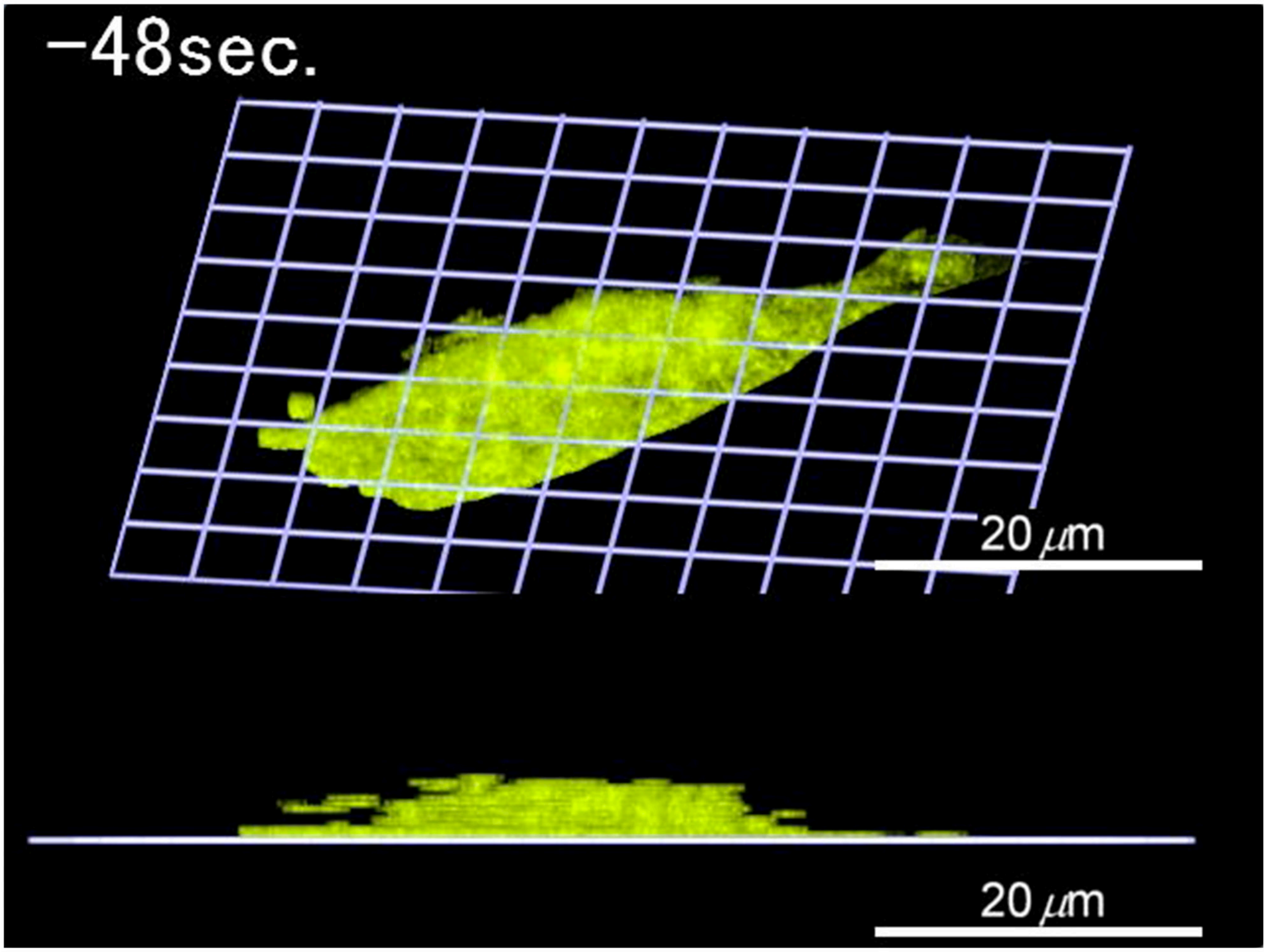

Supplementary Video 3: Volumetric movie in 3D LC-QPM with continuous NIR light exposure in 50 mM dextran in Tra-IR700 treated 3T3-HER2 cell.

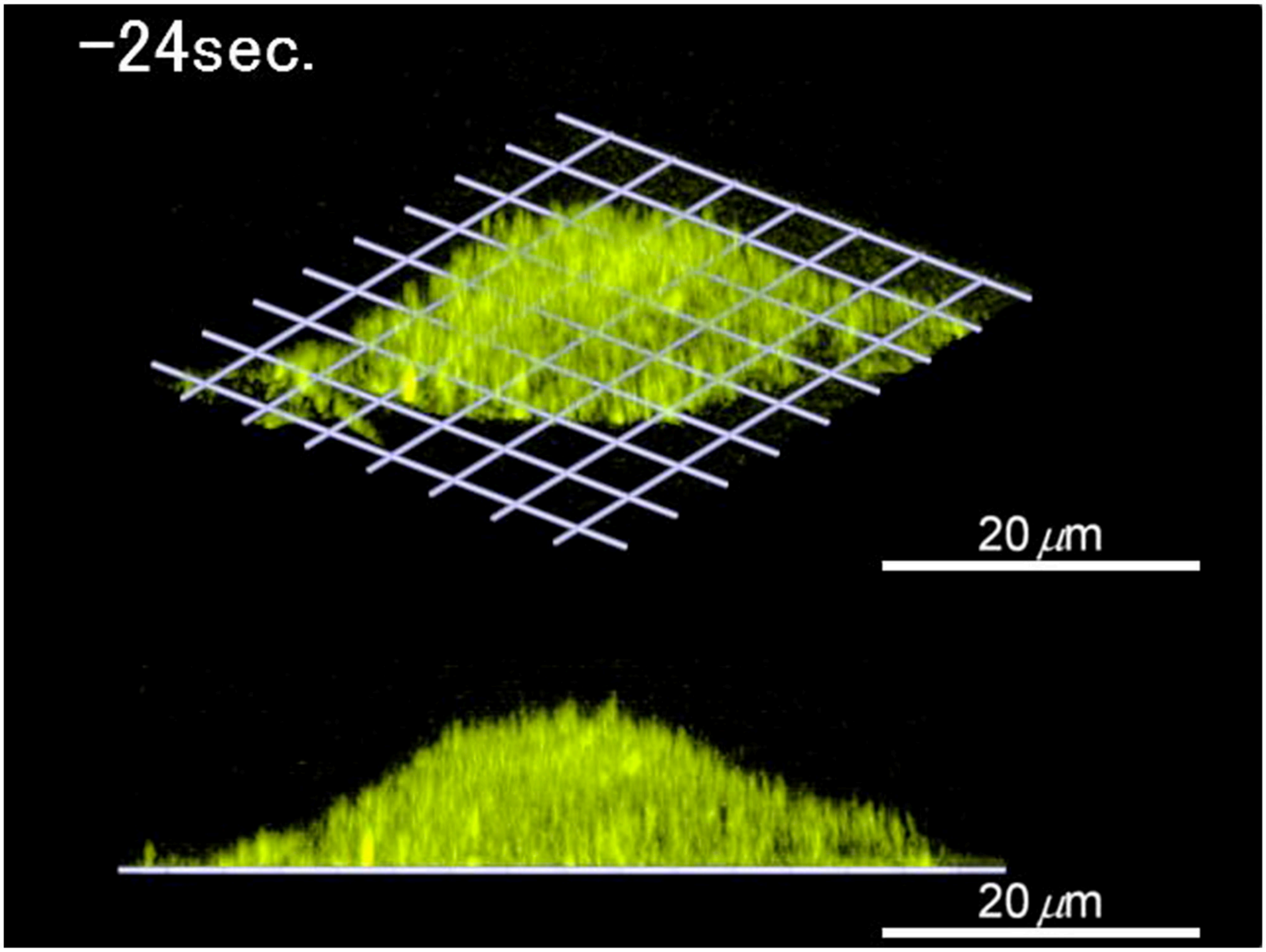

Supplementary Video 4: Volumetric movie in 3D LC-QPM with continuous NIR light exposure in Tra-IR700 non-treated 3T3-HER2 cell.

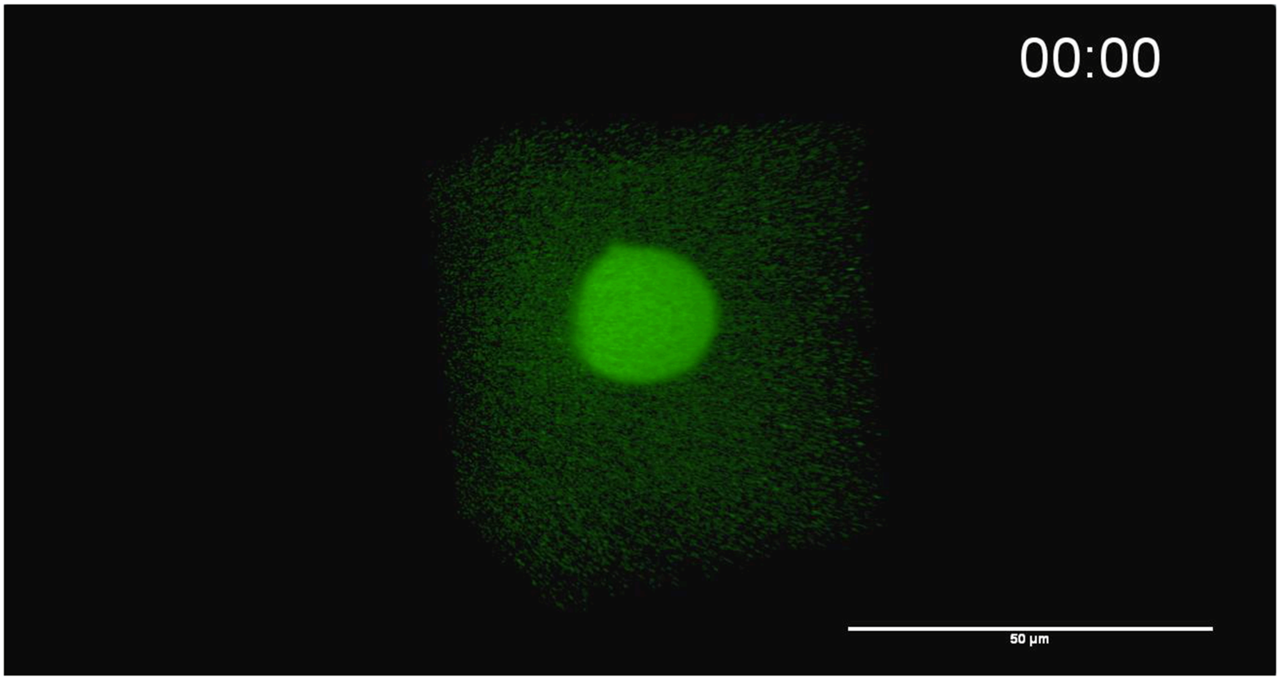

Supplementary Video 5: Volumetric movie was acquired with diSPIM, and demonstrates the result of NIR-PIT in Tra-IR700 treated 3T3-HER2 cell. The cell began to swell shortly after exposure to NIR light excitation and burst during observation.

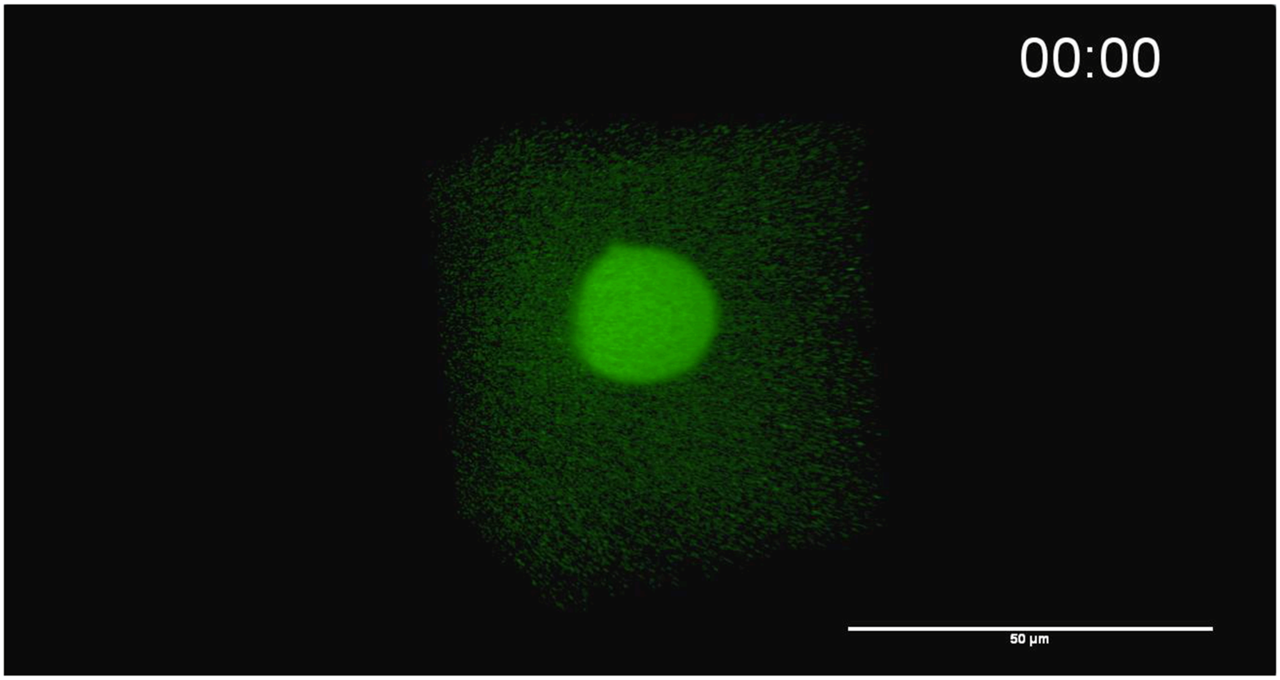

Supplementary Video 6: Volumetric movie was acquired with diSPIM, and demonstrates the result of NIR-PIT in Tra-IR700 treated 3T3-HER2 cell. The cell began to swell shortly after exposure to NIR light excitation and continued swelling even 15-min after exposure of NIR light.

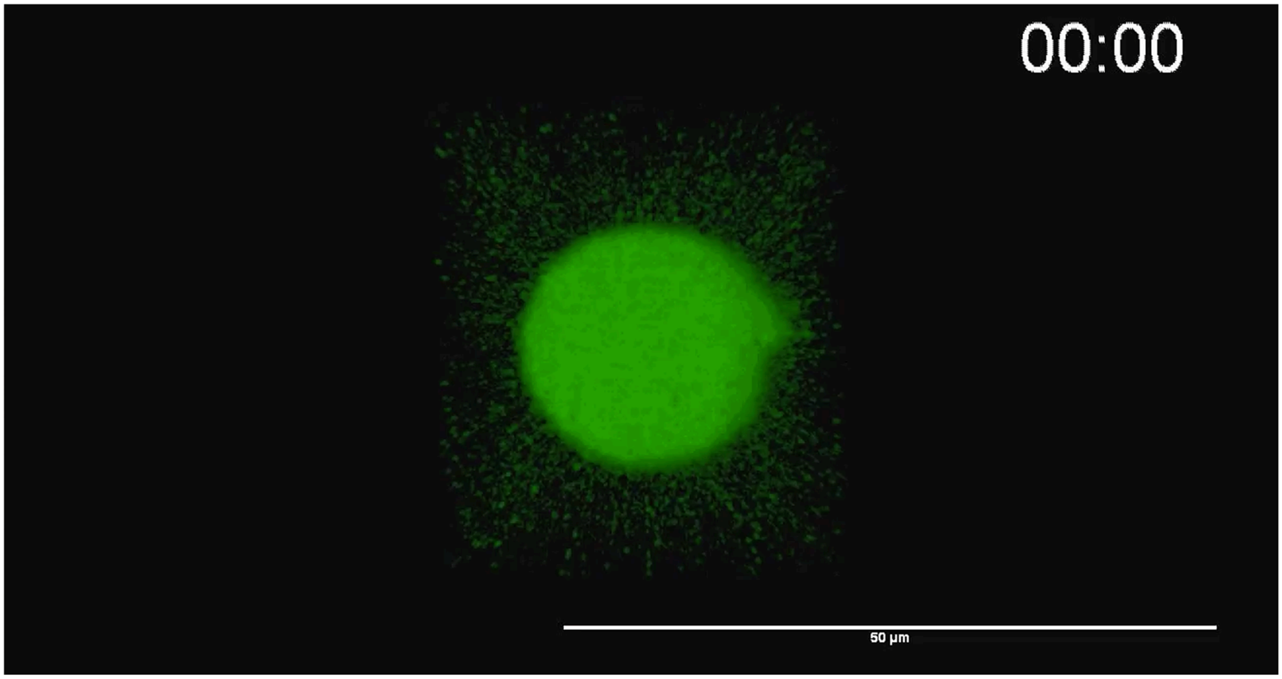

Supplementary Video 7: Volumetric movie was acquired with diSPIM in Tra-IR700 non-treated 3T3-HER2 cell with no NIR exposure. The cell did not show swelling.
